# Healthcare in a system of punishment: how clinicians experience care work within carceral settings

**DOI:** 10.1186/s40352-026-00399-z

**Published:** 2026-02-19

**Authors:** Natalie Keller, Andrew Sudler, Leslie Riddle, Jennifer Elyse James

**Affiliations:** University of California, San Francisco, San Francisco, USA

**Keywords:** Moral distress, Burnout, Dual loyalty, Incarceration, Healthcare, Clinicians, Prison, Jail, Qualitative research

## Abstract

**Background:**

The harms experienced by individuals incarcerated in prisons and jails have been well-documented; however, little is known about the experience of healthcare clinicians in their work in carceral institutions. Using qualitative methods, we interviewed 20 clinicians who deliver care in California’s prisons and jails to better understand their experiences.

**Results:**

Participants described a carceral culture which impeded the delivery of care. These barriers to providing healthcare included limiting the clinicians’ ability to develop a trusting patient-clinician relationship and structural limitations which prevented clinicians’ capability to deliver care informed by their expertise. Participants also described forms of institutional control that affected clinicians on a personal level and in their provision of care. We found that clinicians often attempted to advocate on behalf of their patients; however, clinicians struggled to balance their dual loyalties to patients and the carceral system. This often led them to experience moral distress which ultimately led to burnout. These experiences resulted in clinicians developing self-protection strategies by accepting parts of the system, leaving the system, or continuing to try to change the system.

**Conclusions:**

The barriers to providing healthcare described by participants are inherent components of prisons and jails, which the clinicians had to adapt to in order to treat their patients. Findings from this study demonstrate the system-wide impacts of the punitive nature of carceral institutions which can even permeate the delivery of healthcare. These findings emphasize the need for additional research examining the impact of carceral culture on clinicians and the care they deliver in carceral facilities.

## Introduction

The US has one of the highest incarceration rates in the world, with more than 7 million people cycling in and out of prisons and jails each year (Prison Policy Initiative, 2025). Due to the fragmented care available in community settings, prisons and jails often operate as de facto medical and psychiatric safety-net systems for incarcerated people from impoverished and marginalized communities (James et al., 2023; Sufrin, 2017; Sharp, 2018). However, the actual health services that incarcerated people are entitled to remain open to legal interpretation (Alsan et al., 2023). Colloquially the 8th amendment is often referenced as entitling incarcerated people to healthcare, however, in practice, rather than guaranteeing a positive right to healthcare, it guards against “deliberate indifference” to serious medical need (Alsan et al., 2023). While incarcerated people do have access to healthcare services, they still experience poor health outcomes in comparison to the general population. Compared to the free population, incarcerated people have higher rates of infectious disease, substance use disorders, and psychiatric and chronic illnesses (Caldwell et al. 2001; Genders et al., 2016; Leigey & Hodge, 2012; Nijhawan et al., 2010; Nowotny et al., 2017; Loeb & Steffensmeier, 2011). These disparities are present before, during, and after incarceration (Binswanger et al., 2009). While our knowledge about the health of the incarcerated population has increased over the years there is still a need for additional research.

There is a vital need for additional research examining the health of the incarcerated population, and even less is known about the health and well-being of people who work inside prisons and jails. The exact number of clinicians working in prisons and jails is unknown; however, a 2014 report documented more than 3,200 healthcare workers in the federal prison system alone (U.S. Department of Justice, 2016). The population in federal prisons makes up only 7% of the total number of incarcerated people in the US (Prison Policy Initiative, 2025), meaning that the total number of healthcare workers in carceral settings is likely in the tens of thousands. While much has been written about working conditions in community-based clinical settings, particularly during times of crisis like the COVID-19 pandemic, less has been studied about healthcare workers in prisons and jails.

A recent scoping review of articles on the provision of correctional healthcare identified 23 papers published between 1987 and 2017, of which seven of the articles were based in the US (Simon et al., 2020). Most of the articles incorporated multiple health disciplines, with the majority focused on nursing and psychiatry, as well as an emphasis on teaching and training in correctional medicine. Of the 23 articles eight were descriptive, eight utilized qualitative methods, and seven were based on cross-sectional surveys. The review identified several major themes experienced by healthcare providers including challenges of the distinctive working environment, burnout, safety concerns, and the presence of ethical dilemmas, including the tension between security and clinical considerations (Simon et al., 2020). The findings of the review suggest that clinicians in carceral settings seem to require a specific skill set to deliver care, with several articles finding that clinicians did not feel that they were able to provide the same quality of care in carceral settings as they could in community settings (Simon et al., 2020). However, the authors note that the literature examining perspectives of clinicians who deliver care in carceral settings is very limited, especially for studies based in the US considering the high rates of incarceration in the country (Simon et al., 2020).

This tension is commonly known as dual loyalty, an ethical dilemma often encountered by healthcare professionals, which may be defined as a clinical role conflict between professional duties to a patient and obligations, expressed or implied, and the interests of a third party such as the prison administration or state authority (Pont et al., 2012). While dual loyalty can occur in many settings, it may be particularly pronounced in prisons where the systemic prioritization of security and punishment can exacerbate this tension. In “Examining Carceral Medicine Through Critical Phenomenology”, Andrea Pitts argues that “medical providers are not sufficiently able to carry out their duties to provide caring, therapeutic, or ameliorative encounters with their patients while operating under the punitive aims of jails, prisons, and detention facilities” (Pitts, 2018:15). This tension can also lead to another concept known as moral distress which was first defined in nursing literature in 1984 by Jameton (1984) as the negative experience that occurs “when one knows the right thing to do, but institutional constraints make it nearly impossible to pursue” (6). These challenges to delivering care in carceral settings impact clinicians with one study finding that psychologists working in prison and jail settings experience higher levels of burnout and lower job satisfaction in comparison to psychologists working in other settings (Senter et al., 2010). Little research has explored moral distress or burnout among clinicians working in carceral settings (Smith et al., 2021).

In this paper we draw from interviews with 20 clinicians working in prisons and jails in California to better understand the experience of providing care within carceral institutions. We examine themes that emerged from these interviews with a focus on the barriers to care that result from carceral culture. We examine how these barriers shape the provision of healthcare and how they impact clinicians and their patients. We also aim to advance a new understanding of healthcare labor within the prison industrial complex.

## Methods

This paper draws from two studies of healthcare in prison and jail settings in California. The first focused on experiences living and working in prisons and jails during the COVID-19 pandemic and the second focused on reproductive healthcare in prisons and jails. Each study, conducted independently but by the same research team, involved interviews with formerly incarcerated patients, clinicians working in prison and jail settings, and other experts and advocates knowledgeable on the topic. Findings centered on patient experiences have been published elsewhere (James et al., 2023; Avila & James, 2024; Elster et al., 2025). Here, we focus on the clinician experience. Through parallel data analysis of separate datasets, we observed that similar themes were emerging from clinician interviews. We engaged in additional focused coding of the two datasets with the goal of developing a comprehensive understanding of the experience of providing healthcare in carceral settings.

For both studies, clinicians were recruited via snowball sampling with the help of community organizations and partners, as well as direct outreach to those in our personal and professional networks. The sample size was largely driven by sampling strategy and the availability of the respondents. We aimed to recruit participants who worked across several disciplines and institutions. The small sample size is appropriate, given the nature of this exploratory, qualitative research and our aim of exploring each participant’s narrative in depth and enabling significant reflection, dialogue and time on each transcript.

In total, from 2021 to 2023 we conducted semi-structured interviews with 20 clinicians via Zoom or phone which lasted 1 to 1.5 h (Table 1). Interviews were audio-recorded, transcribed verbatim, and analyzed using a grounded theory framework (Corbin & Strauss, 2008) to allow for an inductive and data-driven analytic process. Researchers began data analysis by engaging in a process of open coding (Bryant & Charmaz, 2007) to create an initial set of inductive codes grounded in the data and deductive codes based on prior research with similar populations. Researchers discussed individual lists of codes and worked collaboratively to develop a code list that was continually adapted over the course of analysis. Researchers then engaged in independent parallel coding of each transcript using the qualitative analysis software Atlas.ti and met regularly as a team to discuss any discrepancies in coding and come to a consensus on codes and definitions. Reports were generated for each code, and the quotations associated with each code were then further refined into categories and then into themes in an iterative process. These themes were discussed in-depth in research meetings. Our team included diversity of race, ethnicity, and discipline, including both social scientists and a clinician who has worked in carceral settings, allowing us to approach the data from many perspectives.

**Table 1:** Participant demographics

Demographics	*N* = 20	%
Age		
20–29	1	5
30–39	8	40
40–49	4	20
50–59	6	30
60–69	1	5
Race/Ethnicity		
White	16	80
Asian, Pacific Islander, South Asian	3	15
Black	1	5
Gender		
Female	17	85
Male	3	15
Discipline		
Nurse	4	20
Mental Health Clinician	2	10
Physician	9	45
Physician with Leadership Position	5	25
Years Working in Correctional Health		
1–5	8	40
6–10	6	30
11–15	4	20
16–20	2	10
Type of Carceral Facility		
Prison	8	40
Jail	12	60

“Physician with leadership position” includes supervisor, chief physician, and director roles

Both studies were approved by the University of California, San Francisco Institutional Review Board. All participants provided verbal consent to participate. This research was conducted in accordance with the Declaration of Helsinki.

## Findings

Our findings are largely organized around two larger themes: how carceral culture produces barriers to care delivery and clinician responses to barriers to care. We will first outline carceral culture through three interconnected examples that result in barriers to healthcare. The first barrier, the “inmate patient” framework, describes how clinicians view stigmatizing language in relation to care. Next, we will explain how the institutional control embedded in carceral settings extends to clinicians and their work. For the third barrier, we will describe how the structural limitations of prisons and jails impede clinicians’ ability to deliver care despite their best efforts. We will then detail how the clinician-patient relationship is impacted by carceral culture and these barriers. Finally, we will examine some of the ways clinicians responded to these barriers.

### Carceral culture

**Fig. 1:**
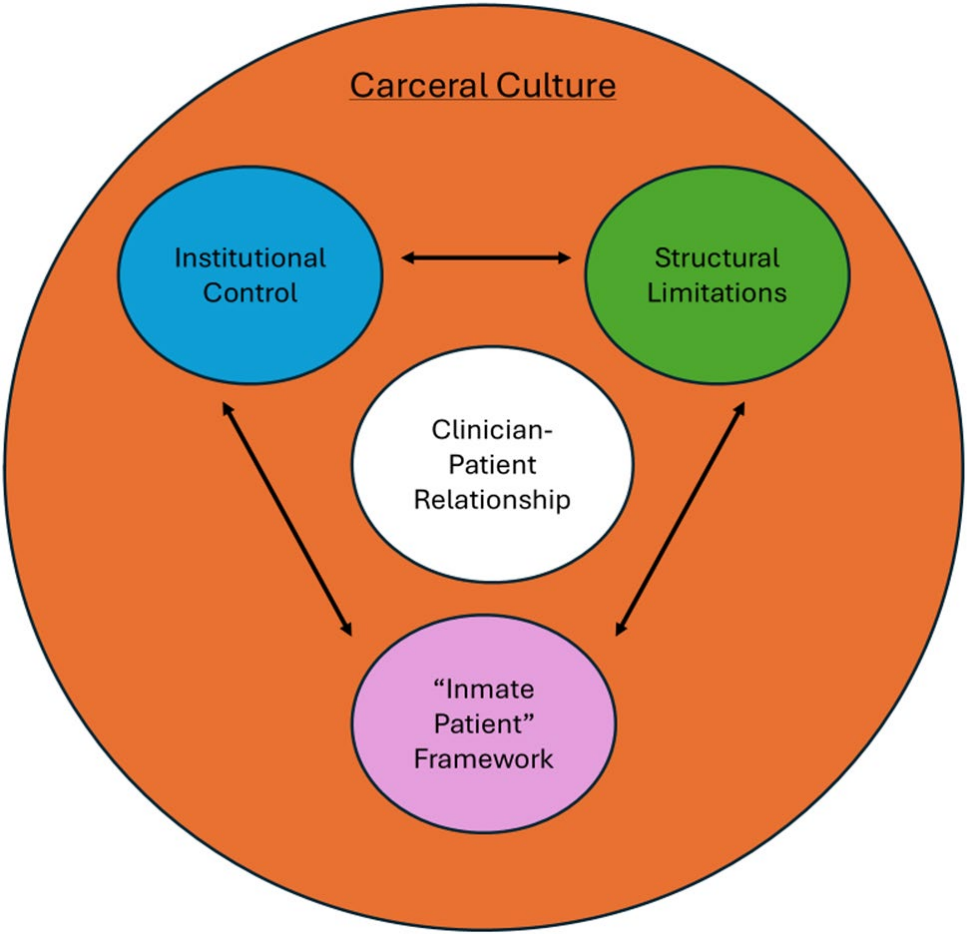
Levels of barriers to healthcare

Clinicians described a carceral culture that permeated clinical interactions. While the participants we spoke to came to this work for a variety of reasons, many participants included mission-driven reasons as part of their current motivation for continuing this work. Yet, many were unprepared for the ways in which carceral culture and the structural realities of the prison environment would shape the delivery of care. Despite their best intentions toward creating a caring and healing environment, the culture of carcerality was pervasive in the institution and disrupted their work, creating unique barriers to care. We offer three interconnected examples of carceral culture that create barriers to care (Fig. 1).

#### Critical components of the carceral culture

When we asked clinicians about their work in prison and jail settings, most would offer a refrain that was something akin to “you know, it’s jail” (P13) or “it’s different when you’re in prison, right?” (P8). There was an unspoken (or not fully articulated) shared understanding that there was a different culture in carceral settings, and that while most clinicians attempted to provide a consistent level of care, inside and outside of the institution, their work environment was starkly different from community-based settings in tangible and intangible ways. We came to see the culture of the prison as a key determinant in the delivery of care inside. The punitive nature of the carceral setting, including its primary mission of safety and security, permeated the medical units. Clinicians felt that this often took precedence over the health of the incarcerated population and the provision of care. Clinicians described an institutional assumption that all patients were dangerous or had negative intent, which influenced if, when, and how patients could access care. The carceral culture, based in discipline, punishment, and control, was also directed at clinicians, with clinicians expressing concerns that their actions were surveilled and their motivations questioned.

#### "Inmate patient"

Clinicians referenced a culture antithetical to caring in which incarcerated people were viewed as “inmates” before patients. The theory that language shapes our ideas and therefore has the potential to impact society has long informed efforts to encourage use of person-first language. Link and Phelan’s (2001) conceptualization of stigma theorizes that when people’s differences are labeled they are associated with negative stereotypes and used to separate those who are stigmatized from those who are not, ultimately leading to loss of status and discrimination. As a way to avoid stigmatizing language when referring to those with a history of incarceration, advocates of person-first language have promoted replacing language like “inmate”, “felon,” and “offender” with terms like “incarcerated person” and “formerly incarcerated individuals” (Tran et al., 2018; Bedell et al., 2019; Cox, 2020). While some participants used person-first language, other participants referred to their patients as “inmates” and “inmate patients.” A clinician described their perspective on using this language,

I call them residents or patients, but you’re supposed to call them “inmate patients” or “inmates,” right, because inmates are, you know, it’s acceptable, you know, to the custody staff that you call them what they are. - P8 (physician, leadership role).

While this clinician clarified that they do not refer to their patients as “inmate patients”, they shared how using this language is not only normalized but understood as the standard language that clinicians are expected to use when referring to their patients. As this participant explained, “you call them what they are” which can be understood to mean that their patients are first defined by their status as inmates, even when receiving healthcare. This stigmatizing language results in an environment in which clinicians are expected to view their patients as inmates, instead of patients.

Other clinicians explained their reasoning for avoiding this type of language when referring to their patients:

I think it’s more humanizing to say patients because, first and foremost, I am there to treat their mental illness. And, yeah, they’re inmates but I…see most of my patients as people that probably should never have been in prison. They should’ve been in a psychiatric hospital…I feel like a lot of my patients…Because I’ve read their crimes and I see how their mental illness mitigated it or played a big role in it. And so, I don’t know, it feels wrong to just call them inmates to me. - P5 (psychologist).

Here, the participant described how she uses language to maintain the humanity of her patients who she does not believe should be incarcerated to begin with. This participant went on to explain that her preferred language conflicted with the official language of her employer, “They say the technical, the PC term that CDCR would like us to use in documentation if we don’t use patients is IP which is inmate patient” (P5, psychologist). Despite this participant’s effort to avoid referring to her patients as “inmate patients” she explains how this language is normalized to different degrees depending on the department within the prison. Additionally, this participant highlights that clinicians are expected to refer to patients as “inmate patients” in documents about their patients. While participants expressed that the *language* they use to describe their patients is important, the idea of “inmate patient” goes beyond terminology; research has found that people who are incarcerated often feel that when they receive healthcare they are not treated as patients but rather as inmates (James, 2023). This distinction demonstrates how the punitive nature of the carceral environment can permeate through healthcare delivery. The standardization of language like “inmate patient” is emblematic of an environment in which clinicians must perform ongoing emotional labor to maintain the humanity of their patients which can be minimized or erased when defaulting to the institution’s standard language of “inmate patient”.

Another clinician shared how they made an effort to view their patients as patients rather than prisoners:

What I try to bring to this job is to not treat them as prisoners in the sense that I treat them the same as I treated my…patients [in the community]…I know that gets through to them and they appreciate that because they feel respected. So that’s what I mean by sometimes I have to remind myself. Like I just treat them as patients. They’re just my patients. And we have the same conversations I’d like to think that I’d have with anybody. - P7 (physician).

Despite striving to offer the same services to incarcerated patients as they did to their patients in the community, this participant is describing that this is an ongoing process. This is due not only to the standardization of this stigmatizing language, which is intentional to center carceral status even in a medical environment, but to the larger carceral culture of treating patients as an othered group. Person-first language has been noted as an important intervention to reduce stigma and discrimination associated with incarceration (Tran et al., 2018; Bedell et al., 2019; Cox, 2020), however, what our participants described was that even using person-first language was not enough to overcome the structural treatment of patients as “inmates” first by the institution.

#### Institutional control

Clinicians we interviewed spoke of institutional barriers to both their patients’ ability to make choices about their health and their own abilities to work within prisons and jails. Some clinicians expressed frustrations about dealing with the bureaucratic elements of their job. One clinician described the paperwork needed to ensure that patients had proper accommodations for work and housing, access to durable medical equipment, and even prescription medications:

You can’t say [to the patient] ‘don’t climb,’ you have to fill out a form that says…‘you have these limitations in work.’ And another…form if there is a disability and you need a wheelchair. And then you have to make sure you fill out the right orders for getting the wheelchair…So there’s a lot of different things that you have to fill out so they can get what they need…Ideally most of the prescriptions are filled by the central fill pharmacy…but you do have to do a lot more work, because you can’t just say, ‘hey, here’s your prescription, take it to the pharmacy.’ You have to make sure…You’re filling in bunches of little boxes on the computer. It takes a long time to do it all. - P11 (physician).

While filling out paperwork is a part of any clinician’s job, several clinicians we interviewed described time-consuming bureaucratic tasks that were unique to delivering care within prisons and jails and effectively reduced the time that could be spent with patients. Participants described barriers to delivering care and their capacity to work that are not typically experienced by clinicians in the community. For example:

We know for sure that they watch our email. They read our email. There is zero privacy…it’s a culture of control. And they make very little distinction between inmates and staff…As an organization they are into controlling people. That’s it…So the CDCR [California Department of Corrections and Rehabilitation] is an organization that controls people…And I’m a CDCR employee. I don’t get to speak my opinion, I don’t get to have my choice in 99% of the things that I do. - P4 (nurse).

This nurse expressed a lack of agency and a sense of being surveilled and controlled by the carceral institution. Other clinicians also detailed instances when their medical expertise was ignored by correctional officers and staff when prescribing treatment for their patients, echoing this clinician’s sentiment that, even as employees, they often do not feel like they are allowed to voice their treatment recommendations.

Another clinician also expressed how she felt pressured to follow what her employer asked of her in various contexts. This participant described experiencing “anxiety” in anticipation of getting written up because she felt that “if you don’t do everything that they want you to do, you get written up” (P5, psychologist). This included when she was given orders that contradicted her clinical recommendations,

“I’m not telling you what to do,” but you’re being redirected. “I’m not telling you what to do, but this.” And it’s like, okay, so what happens if I refuse? Do I get written up? Do I get walked off? Like I don’t know what the consequences are to not going with what they say. - P5 (psychologist).

This psychologist explained how her employer would be careful not to order her to conform with their directions, but she felt pressured to fall in line, especially due to concerns about the unclear consequences that could result from refusing to conform. While this clinician had been written up in the past and she was concerned about being written up again, she was also worried about experiencing even harsher consequences. The participants’ experiences show how clinicians are surveilled as well as how they are taught to self-surveil to follow the expectations of the carceral institutions where they work. This can cause clinicians additional moral distress as they must learn to balance their responsibilities as clinicians with the looming risk of possible consequences to their employment and livelihood if they do not follow the institutional expectations. While we typically understand that the controlling environment of prisons exists for the purpose of controlling those who are incarcerated, these institutional practices extend to employees, including healthcare workers, as well.

#### Structural limitations

Several participants noted that many of their patients received consistent healthcare for the first time while incarcerated, highlighting how often carceral institutions end up providing a secondary purpose as a health management organization. However, due to prisons’ primary purpose of punishment and security, the healthcare needs of incarcerated people are limited by the very structure of prisons. Despite clinicians’ best efforts to support their patients in healing, they expressed limitations due to the constraints of providing care in carceral settings:

There are limits to what I can do here because I’m at a prison. There are certain medications I can’t prescribe, there are certain medications that can only be given by the nurse at a window that can’t be taken back to the cell. There are certain limitations to resources, you know. There is a two to three-month wait for physical therapy. - P7 (physician).

Similarly, clinicians noted the structural constraints on health beyond what takes place in the clinic. In addition to being limited on medications they could prescribe or treatments they could offer, they were limited in what advice they could give to their patients in terms of things like diet, exercise, or other supports needed due to medical conditions:

Most of the challenges were like, you know, what a patient could have in their cell…Do they need like a double mattress? Do they need a special, you know, something to make them more comfortable during pregnancy etc.?…I mean you don’t have any choice, right? So you don’t have much choice about like what setting you are in. You don’t have much choice about what you are eating, that sort of thing. I can’t imagine being pregnant and not having those choices. - P14 (physician, leadership role).

Several clinicians also highlighted specifically the challenges of wanting to make recommendations related to food but feeling limited in encouraging health outside of the clinic.

When they are incarcerated, they don’t really have control over the food that they get. And also, a lot of times, they don’t feel like they’re getting enough food. And so they tend to eat commissary food. And most of commissary is stuff that is completely unhealthy. - P16 (physician).

While clinicians would typically make recommendations to their patients in the community regarding diet and exercise, participants explained that this was limited for their incarcerated patients who did not have any control over the food they had access to or exercise.

Even when clinicians could or should have more control, some felt that they did not. One clinician described trying to follow proper channels to get necessary resources for the patients, but facing barriers:

Like wouldn’t I love to be able to prescribe a diabetic diet? But they get the diet they get and it’s not diabetic…So, you know, we have great intentions. We order something, it doesn’t go through. Or something doesn’t happen the way it’s supposed to. And that happens a lot. - P7 (physician).

This clinician went on to describe the dire circumstances that their incarcerated patients experienced in the food they were given at the beginning of the COVID-19 pandemic.

There were times there weren’t even meals being served. There wasn’t even food. There was like boxes of onions, whole onions being delivered and that was it. - P7 (physician).

The limitations of healthcare in prisons and jails were particularly evident during the lockdown period at the beginning of the COVID-19 pandemic. While an exceptional moment in history in many ways – indeed, limitations on food, medicines, and supplies were rampant across the globe – the pandemic also laid bare some of the structural limitations on care and sources of burnout that are always present inside carceral facilities, if not typically to the same extreme. Indeed, during interviews about providing care during the pandemic, many participants described barriers to care delivery that were heightened or exacerbated, but not novel or unique to the pandemic itself. Further, COVID-19 outbreaks in prisons and jails were rampant throughout the US due to overcrowding, inconsistent testing, and personal protective equipment shortages (Nelson & Kaminsky, 2020), often mirroring disease outbreaks and health delivery challenges that predated COVID-19. Additionally, the structural limitations of prisons and jails posed a challenge to following public health protocols, such as social distancing, during a pandemic (Hawks, Woolhandler, and McCormick 2020).

Participants described the realities of the pandemic for incarcerated people, with one critiquing the expectation that they could protect themselves by wearing masks: “the inmates don’t have protection. They were given a mask. Which they can’t wear 24/7. So that’s already a problem” (P9, physician, leadership role). Another participant explained that the virus “went through [the prison] like wildfire…they [incarcerated people] are sitting ducks. There was nothing they could do” (P7, physician). While these clinicians described how they did their best to implement public health recommendations by adapting to the carceral settings their patients were incarcerated within, the structural limitations prevented following all recommendations. Clinicians explained how their inability to protect their patients from the COVID-19 virus, due to the limitations of prisons, led to them experience moral distress, “it felt completely abandoned and I felt like all the inmates were trapped in their cells to get COVID” (P9, physician, leadership role). Another participant echoed similar sentiments, “it’s a hard feeling as a doctor. I never felt that helpless” (P7, physician). The COVID-19 pandemic demonstrated that despite clinicians’ best efforts, there are limits to providing healthcare in carceral settings. While some barriers were unique to the pandemic, routinely, medications, mobility devices, personal hygiene, or safety equipment may be limited due to security concerns or the structural nature of the punitive environment. These limitations resulted in clinicians experiencing moral injury when they realized they were restricted in their attempt to protect their incarcerated patients’ health from a rapidly developing global pandemic.

As we will describe in another section, clinicians engaged in many strategies to overcome structural barriers to providing care. However, while some structural barriers had become normalized as a baseline obstacle to providing care in prisons, the COVID-19 pandemic and clinicians’ inability to keep their patients safe from an acute emergency became, for some, a catalyst for moral injury when they were restricted in their attempt to protect their incarcerated patients.

#### Carceral culture and the patient-clinician relationship

The patient-clinician relationship in prisons and jails is limited by the distinct clinical environment which is shaped by carceral culture and the aforementioned barriers. One example of this in the patient-clinician relationship is that correctional staff played a key role in whether and to what extent clinicians could treat patients in accordance with their clinical values. By design, custody staff are a critical aspect of healthcare delivery in carceral settings. In addition to their roles and responsibilities related to safety and security, clinicians rely on correctional staff for patient triage and transport. In that sense, collaboration between clinicians and correctional staff is essential, and yet many clinicians note the tension of both relying on custody and wanting to distinguish between custody and clinical care:

You need custody to bring patients to you and you need custody to bring patients to the hospital. And just like to help provide care you need custody’s eyes and ears to see how patients are doing. You need to work as a team *and* you need to be really separate. - P14 (physician, leadership role).

This clinician is describing how correctional officers can support clinicians by keeping them informed about their patients. However, clinicians also had a desire to maintain a separation and to operate independently from the correctional goals of the institution. The needed reliance, coupled with the power and authority that correctional officers have, leads clinicians to perceive officers as acting as gatekeepers to care; an officer’s perception of severity of illness or a security concern can impact if or what kind of care is available to the patient:

We have a list, we tell the custody officers that we need to see this and this and this person out of their cell and the custody officers usually will tell us, “this person is very aggressive today, I don’t think she should come out unless you really feel like she should come out”…Most of the time, either they refuse or we see them. - P12 (physician).

This created a complex dynamic in which both patients and clinicians depend on custody staff to access healthcare interactions. Additionally, as noted in both of the previous quotes, clinicians counted on correctional staff to communicate about how their patients were doing. However, as described by P12, the correctional staff’s security concerns could be prioritized over health concerns. While this clinician described that they usually see patients despite these types of warnings of “aggression,” other clinicians may or may not, subconsciously, be primed to view patients differently based on information shared by correctional staff. Many clinicians described both wanting to establish firm boundaries between their work and the work of correctional staff but also experiencing tension to what they saw as a misunderstanding of the custodial obligations of carceral institutions. As one physician described:

It requires being at odds with the system that you’re working within, right? I am not part of the Sheriff’s Department, they are not my colleagues in providing care…the legal authority to care for a patient in many ways trumps their responsibility to keep this person confined…what I have to remind people a lot is, their job is not to keep that person necessarily confined, but to keep that person safe. That’s what custody means, right? You have to limit their ability to escape, but you also have to limit the harm, prevent harm that could happen to them. - P18 (physician, director-level position).

This clinician describes the tensions experienced by healthcare workers within a carceral system in which they have to balance the priorities of the carceral system, a system whose main priority is security, while also prioritizing caring for their patients. They are also asserting that they are not a part of the correctional staff team; they see their role as distinct and, as another physician described, expressed concern that the assumed allegiance between clinical and carceral staff can challenge the ability of clinicians to develop trust with their patients:

I mean the instinct [for incarcerated people] is to not trust anybody when you’re in jail…Like of course you wouldn’t trust anyone, you’re incarcerated. So we had to be very, very separate from custody. That had to be very, very clear that any information you gave to us we were not going to share with custody. And this was a constant line that needed to be drawn and reinforced…But that line was always challenging. - P14 (physician, leadership role).

Delineating boundaries between healthcare and corrections was not always easy; most clinicians acknowledged that despite their best intentions, they were inherently part of a system that is contrary to health and inhibits their medical practice. Clinicians must maintain a careful balance of being trustworthy in the eyes of their patients—which involves distancing themselves from the correctional goals of the institutions—while also relying on corrections to facilitate transportation to care and even act as first responders when emergencies arise outside of clinical spaces. One clinician described this tension, saying,

It is an interesting and weird place as far as forming relationships [with patients]. It’s kind of like you’re in some sort of complicated polyamorous relationship where the other person in the relationship is abusive and it’s the sheriff’s department…When you’re in a setting where the ethos is kind of contrary to you being happy and healthy and then you’re spending time around someone whose goal it is to help you connect with what you need to be happy and healthy, I think that can feel very positive…Like yesterday I was going to see a patient…and I can hear deputies just accosting somebody…while I’m trying to just be in the mental space to see this person and talk to them…sometimes it’s hard to feel therapeutic when you’re in a really untherapeutic space. - P15 (physician).

This clinician is describing both the dual loyalty—being in the middle of a relationship between patient, clinician, and jailer—as well as the structurally violent nature of the institution. Importantly, she is describing how the system is not only violent for patients, but how this violence extends to her care work by disrupting her ability to engage in an effective therapeutic relationship. Witnessing this harm results in a moral injury which disrupts the clinician’s ability to engage in care with her patient. Yet, this creates an inherent dilemma. Can one be loyal to both an individual and their captor? If the goals and values of the institution and the individual are inherently in conflict, to whom is the clinician aligned?

Regardless of whether or how a clinician aligns with the institution, many reported that their role as an employee of a carceral institution disrupted the possibility of building bidirectional trusting relationships with their patients. While in all clinical settings the patient-clinician relationship has been found to benefit from trust, it is also considered to be fragile due to the unequal power dynamic (Delmar, 2012). The unequal power dynamic inherent to the clinician-patient relationship is exacerbated by the additional vulnerability of being incarcerated while receiving healthcare (Fuller & Eves, 2017) and the structural realities of care in carceral settings.

When examining the experience of delivering care within carceral settings it is necessary to include the context of the punishing environment in which clinicians are meant to provide care. Providing healthcare in a prison, where the purpose of being incarcerated is to be punished, can be challenging for clinicians whose goals are to heal and care for patients (Weiskopf, 2005). Due to the punitive nature of prisons and jails, Storch and Peternelj-Taylor (2009) argue that clinicians could become used to the punishing environment and become desensitized to the dehumanization of their patients.

One clinician noted that even acknowledging her patients’ humanity was discouraged in this setting.

There are times where you’re sitting with your patient and a terrible story has been told or something really vulnerable has been shared and they might want a hug or they might, you know, need just like a hand to hold. And that wasn’t allowed in jail. You’re not allowed to have physical contact. - P16 (physician).

At the time of the interview, this clinician was no longer working in the jail, noting that burnout had led her to leave so that she could recuperate from the stress of the job and consider other clinical careers. This clinician’s experience demonstrates the challenges of attempting to provide healing care while operating in an environment which discourages clinicians from treating their patients as humans with human needs.

### Clinicians’ response to barriers

**Fig. 2:**
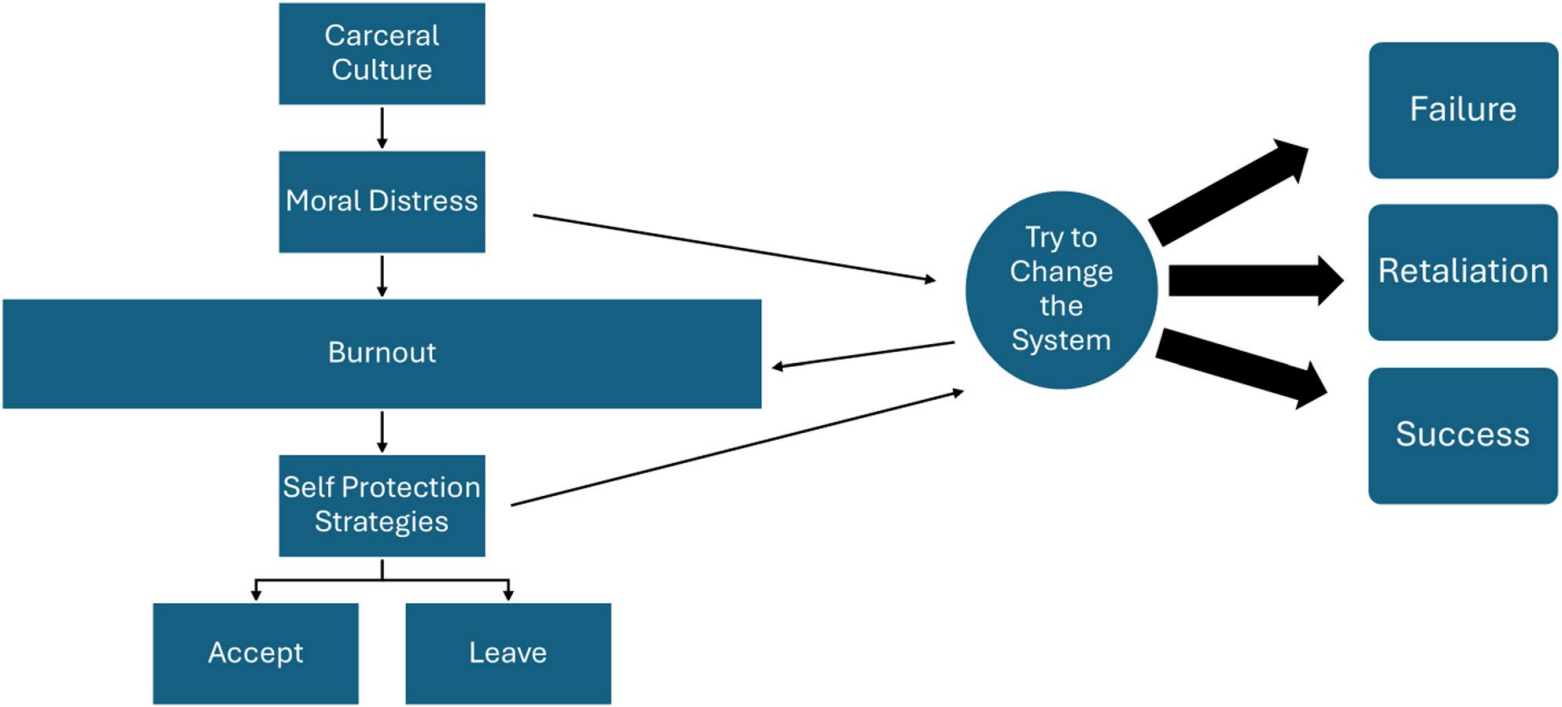
Clinicians’ response to barriers

The majority of the clinicians we interviewed identified themselves as strong advocates for their patients; they came to this work because of a desire to work with the vulnerable patient populations in prisons and jails and, when faced with structural barriers to care delivery, initially sought to overcome them or change the system. What we found is a cyclical pattern often faced by many clinicians when confronting structural barriers to care (Fig. 2). Clinicians described feeling moral distress from the barriers that resulted from carceral culture. This led some to feel burned out, while others were led to try to change the system. This advocacy work was sometimes *successful*, though often required clinicians dedicating a lot of time and effort to achieve the goals of their advocacy. Others were *unable to find success* from their advocacy work and some even experienced *retaliation* for their efforts. These efforts often led to greater burnout – even when small victories were won, which was compounded by the burnout from their existing moral distress. This resulted in clinicians turning to self-protection strategies, including finding ways to accept the system, continuing to advocate to change the system, or leaving the system altogether.

One example of the ways that clinicians responded to barriers is from an OB/GYN who was working in a large jail system. He described a story of a memorable experience *successfully* advocating for a patient to be released from jail.

There was somebody who was in jail at like 33 weeks [pregnant] with multiple health issues on a misdemeanor. I said, “why is she in jail?” And they’re like, “well, there seems to be some miscommunication with the DA”…it’s public record who this DA is, so I found his email and I started sending him emails being like you need to let this person out of jail…I’m not providing you any information except that, as a doctor, this person should not be in jail right now…I wrote that note on a Thursday. I was really hoping she was going to get out Friday but she got out Monday. So it’s like, okay, I don’t know how much it affects but I think there’s a lot of power behind our profession and a lot of power that’s not utilized. - P18 (physician, leadership role).

This clinician is describing taking up advocacy in a way that falls outside of the typical clinical role. This clinician sees advocacy as a part of the “power” of his profession. He feels a professional obligation to advocate for his patients, even in areas outside of his direct medical expertise, when he thinks it will ultimately impact their health and well-being. However, he notes that many other clinicians do not advocate beyond their clinic or institutions, and he also notes he is not always clear if it makes a difference. Yet he felt an obligation to take whatever action he could for the health of his patient, which, during incarceration, often means engaging with the criminal legal system.

This type of advocacy work is not atypical in safety net settings. Both inside and outside of carceral facilities, clinicians advocate for their patients. For many patients, their core needs, the things most impacting their health and well being, may not be captured in the primary diagnosis or solved in the clinical encounter. Many clinicians working in “mission-driven” settings see this type of extra-clinical advocacy work, done to either change systems and structures or to help their patients navigate them, as a critical responsibility of the job. This physician is describing that not all clinicians are utilizing the power they have to support their patients.

I think that’s really hard for a lot of doctors…where efforts that you’re putting in are futile and can result in burnout and it can result in dismissing patient’s needs…the most rewarding care is when you don’t see railing against the system as futile. Where you see some benefit to doing that…if you can take that extra step to meet the needs, then that can be very rewarding and really fights burnout…the things that give me solace are the times where…this patient asked me for something and I was able to provide it. - P18 (physician, leadership role).

This clinician is describing a sentiment we heard across many interviews: clinicians feel that advocacy is a critical part of their jobs, but over time it can lead to burnout and poor care. For this clinician, seeing successful results from his advocacy countered his feeling of burnout. Yet, he was also quick to note that these successes can be rare, making the advocacy seem futile and the possibility of burnout high.

Another OB/GYN who had previously worked at a county jail shared a story of a time when she tried to take a holistic approach by advocating to improve her patients’ access to more nutritious food but was *unable to find the success* she was hoping for.

I kind of worked with their like kitchen staff and the nutritionist who they recently hired to kind of modify their diet…that was also quite challenging, because even with the nutritionist on their staff, there’s like really only so many modifications that they will allow…most of the diets were…not very nutritious…And so I think it was easier to make changes from a medical perspective, but things like getting people more exercise or changing their diet proved to be a lot more challenging. Even though those are things that were certainly related to their health. - P19 (physician).

Despite this clinician’s best efforts to improve the food that her incarcerated patients had access to she found that they were limited due to what is allowed by the prison. This participant ended up feeling that she had more control within the clinical setting than she did in her efforts to encourage health outside of the clinic while still in the prison. This experience led this clinician to accept the system by re-focusing her efforts on the clinical setting instead of advocating towards more systemic change. She later left the system and founded her own clinic where she provides healthcare services for patients with a history of incarceration.

Clinicians are not only battling the systems to provide care, but often their own coworkers, particularly correctional officers. As described earlier, some clinicians felt that correctional staff could create barriers to care. One physician noted the positionality of white clinicians in carceral spaces:

When I talk to medical students I’m like, especially the white people in the room…“embrace your inner ‘Karen’ here…Ask to speak to the manager”…It’s how “Karens” have retained their power throughout all of these years. And so you can use that to benefit your patients in so many ways. - P18 (physician, leadership role).

This physician is describing the power that white clinicians – and in some ways, specifically white female clinicians – have and encouraging them to harness this power when advocating for their patients. Unfortunately, some clinicians also described facing retaliation from correctional staff as a response to their attempts to change the system, often in ways that were sexist or racist. One of the clinicians we interviewed had been a physician at two large prisons. This physician described an instance when she reported officers who were not wearing masks at a time when it was required. However, she shared that she experienced *retaliation* as a result of this advocacy:

Then they [correctional officers] refused to unlock the door…We had like a fire door that has a…big key that only officers [have]…And they didn’t unlock that for a couple of weeks. So I couldn’t go out that way…I always keep the most recent data, like the inmate deaths and the staff deaths on my desk, on the outside of my door. And that got ripped down every week for a while. For months actually. - P9 (physician, leadership role).

This physician explained that she did not feel aligned with the culture and politics of the majority male and conservative environment of the institution she worked in, and felt a particularly gendered dynamic when attempting to advocate as a woman in a predominantly male space. She described feeling at odds with the officers in this prison who were majority male, conservative, and opposed to some aspects of healthcare, including vaccines. While not all clinicians experienced retaliation as a result of their advocacy, this participant was retaliated against by officers. Although white clinicians have power, clinicians who are white women are still vulnerable in this male-dominated space. The retaliation experienced by this physician resulted from her attempts to enforce rules that had been put in place to protect the health of everyone in the prison, including those who were incarcerated as well as staff. She was also retaliated against for maintaining health information, but she accepted the situation and approached this retaliation with an optimistic attitude by seeing it as an opportunity to post updated data when it was ripped down.

An OB/GYN who delivered care in jail settings described the process of her advocacy work to change the food her incarcerated patients were provided.

They told me like you’re not going to be able to change the food menu at the jail. Like it’s been the way that it’s been forever. No, I just emailed people multiple times and harassed them and finally they had to listen to me…It took…probably a year, to actually change the menu…But it was a lot of effort. And I think that it’s just hard to exert that much effort all the time. And I definitely want to continue being an action-oriented person, but…it’s just about like truly choosing your battles. Because I want to believe that you can win every battle, but I think that the self-sacrifice is probably not a good thing. - P16 (physician).

This physician highlighted the importance of advocacy as well as the possible detrimental effects of engaging in advocacy work which requires a substantial amount of time and effort. Additionally, the physician describes how she has to limit her efforts by choosing which “battles” to engage in, as to not sacrifice too much of herself. However, even with a self-protection strategy like this one, clinicians must still make difficult decisions in deciding where to focus their time-consuming efforts.

#### Self-protection strategies

Of participants who were still working in carceral settings at the time of the interview, they expressed a range of feelings about the healthcare provided by the system. Some participants expressed a strong sense that their institutions provided excellent care or better care than would be available to patients if they were not incarcerated. Yet others really struggled with continuing to work in a system that they felt harmed their patients, either through limitations in medical care or by virtue of the system itself.

Several clinicians we spoke to shared their perspectives on the complexities of working within the carceral system with the ultimate goal of providing quality care to their incarcerated patients. These clinicians’ sentiments were often complicated by a feeling of “complicity” despite their best efforts to deliver necessary healthcare. One clinician described this tension by explaining that “the care is respectful but then you know you’re still operating within the system and so it can be complicated” (P15, physician). The participant in the following quote echoed similar sentiments of wrestling with his positionality within the carceral system,

You are within the jail. There is no way that you’re not part of that system. And you have to acknowledge it and you have to like say “this is fucked up and I know that.” And then on the other side you have to say, “but I’m not, you know, I’m not your jailer, I look like him and I have authority and so I understand what I represent, but I will work with you to provide you the care”…those two contradictory things have to be very present…Because if you ignore the fact that you are working within such a system, then you’re also doing a disservice to your patient. - P18 (physician, leadership role).

While many clinicians explicitly pursue jobs in carceral institutions for mission-driven reasons, they often find that their mission is at odds with the fundamental design of prisons and jails. Some of the clinicians described the care that was provided as substandard, even though they approached the job with all the passion and professionalism they could. Some clinicians end up finding that despite their best efforts to change the system from within they are still participating within this system. This creates a profound moral injury; how does one deliver care if they are, in essence, a part of the system that harms? At the time of these interviews clinicians P15 and P18 were continuing to deliver care within the carceral system, ultimately making the decision to stay and accept certain aspects of the system in order to continue this work.

In contrast, another physician engaged with the complexities and limitations of wanting to change the system from within, and her own ultimate decision to step away from practicing medicine inside carceral facilities.

Part of the reason why I also ended up leaving my job is because I was thinking…I’m fighting the system of oppression from within and…I’m doing a good thing, I’m fighting a system of oppression. I’m like one of the good guys. And then all of a sudden I realized, actually, if I’m working within the system of oppression and I am saying I’m an abolitionist but my paycheck is coming from the prison industrial complex because I’m working for this for-profit healthcare corporation, then I am also the oppressor. And that’s when I realized I needed to take a step away and reevaluate like how do I actually provide care to incarcerated folks without being part of the system of oppression? - P16 (physician).

While this physician was able to see some successful results from her advocacy work, she grew to feel a sense of complicity with the carceral system that she did not feel was reduced through advocacy. As a result, this clinician decided to leave the system.

## Discussion

It has been established in the literature that prisons create a challenging environment for clinicians to deliver care in (Simon et al., 2020). However, most of this literature examines prison systems outside of the US and little research has sought to describe the ways in which clinicians seek to challenge structural limitations on care delivery. In this paper we presented a framework to describe how the “inmate patient” label, institutional control, and the structural limitations of prisons and jails together create a carceral culture that prioritizes security and punishment over care delivery. We argue that carceral culture creates a significant barrier to care that disrupts the patient-clinician relationship. This framework of carceral culture becomes a heuristic for understanding how policies, rules and regulations may impact or impede healthcare interventions in carceral settings.

We argue that these structural barriers to care harm not only patients, as has been widely described in the literature (Lupez et al. 2024; Kuhlik and Sufrin., 2020; Fisher & Hatton, 2010), but, and particularly through attempts to improve care delivery or advocate on behalf of individual patients, become a both a source of moral distress and burnout for clinicians themselves.

Carceral institutions have long been considered antithetical to health and wellbeing. As Nancy Dubler notes in *Ethical dilemmas in prison and jail care*,

The mission of medical care is to diagnose, comfort, and cure; the goal of a prison or jail is to confine, punish, and, almost accidentally these days, rehabilitate. There is an inevitable collision between these two sets of goals, exacerbated because correctional facilities are inherently coercive institutions that for security reasons must exercise nearly total control over their residents’ lives and the activities within their confines (Dubler, 2014).

The clinicians in our study felt this collision viscerally. For many, their goal of providing quality healthcare to a vulnerable population had led them to working in carceral systems. Yet, they encountered structural barriers that challenged their understanding of the mission of medicine. Our study found that even the language that clinicians used when communicating about their patients was controlled by the carceral institution, with some clinicians sharing that they were not allowed to use person-first language. However, even those clinicians who did insist on referring to patients as “patients” described that the larger cultural mandate of prioritizing security over patient care remained. This phenomena is supported in the literature with one study, which examined the impact of mandates requiring person-first language in prisons, finding that these mandates did not have the intended effect on how incarcerated people were treated by prison staff (Kushmerick-McCune et al., 2024); rather what is needed is a cultural shift in how incarcerated patients are viewed and structurally relegated within the system (Kushmerick-McCune et al., 2024). We argue that controlling the language that clinicians use is representative of a larger goal of the carceral institution to ensure that carceral culture is present in every area of the prison. In this way, language can be understood as a marker for how carceral institutions attempt to control patient treatment to ensure that the punitive environment extends throughout the entire carceral system, including where the clinical encounter takes place.

Our study found that structural limitations and institutional control were components of the overall carceral culture, which shaped the environment in which clinicians deliver care to incarcerated patients. These barriers impact how clinicians can deliver care to their incarcerated patients and the overall patient-clinician relationship. Despite a common institutional norm of “priming” clinicians to be cautious of their patients (of violence, manipulation) our participants overall did not feel this way about their patients. Most came to this work out of a desire to work with underserved patients and incarcerated patients in particular. Yet, they described levels of barriers which interfered with their ability to provide care that was in line with their values. This led participants to feeling moral distress (Jameton, 1984) as they aimed to balance their dual loyalties (Pont et al., 2012) to their patients and the carceral system which employed them. Despite limited research exploring how clinicians in carceral settings experience moral distress, our findings align with research which finds that these clinicians experience ethical dilemmas, blurred professional boundaries, and burnout (Smith et al., 2021), along with the need to guard against “dual relationships” with their patients and correctional staff (Bonner and Vandecreek, 2006). However, our study finds that moral distress can also be a catalyst for clinicians to engage in advocacy to improve healthcare for their patients. Additionally, we found that both moral distress and advocacy efforts can, independently or concurrently, lead to burnout as well.

As described throughout this paper, providing healthcare within carceral institutions requires clinicians to adapt in unique ways not required of those who deliver care in community settings. We elucidated several strategies that clinicians undertake in response to the barriers created by carceral culture, as well as the variety of barriers they encountered while attempting to adapt. Further, most of the clinicians we spoke to described varying levels of advocacy on behalf of their patients. Some felt that clinicians had a responsibility to advocate for their incarcerated patients and that other clinicians did not use the power that came with their positionality enough. While the majority of the participants were white, we had an overwhelmingly white female sample, with some women experiencing gender-based retaliation by officers for their advocacy. This resulted in particular complexities in the power that these clinicians had to speak up and also led to many clinicians experiencing burnout from repeated attempts to change the system. Burnout particularly affected those who had intentionally pursued these healthcare settings for mission-driven reasons. This led some clinicians to leave the carceral system as they experienced additional barriers to their mission-driven work.

### Limitations

There are several limitations that should be considered in this study. First, the clinicians who participated in this study may not reflect all clinicians who work in carceral institutions. This is due to the recruitment strategy which included snowball sampling and direct outreach to those in our personal and professional networks. Additionally, many clinicians who chose to participate were willing to tell us about and critique the carceral system in which they work, which may not necessarily reflect other clinicians’ perspectives. Second, the majority of our participants were white and female, which may not reflect the overall population of clinicians in prisons and jails. Third, this study draws on data from two separate studies, each focused on a particular healthcare delivery context. It is possible that interviews conducted with an aim of more broadly understanding the experience of clinical care in prisons and jails, or interviews conducted prior to or with more distance from the COVID-19 pandemic, may have produced different findings. Finally, this study only included clinicians who worked in California; therefore, our findings may not be transferable to other states or countries.

## Conclusion

In this paper we demonstrate that the provision of healthcare is limited by the punitive purpose of the carceral system, which affects not only incarcerated patients, but their clinicians. The institutional control and structural limitations are inherent components of the overall carceral culture that defines prisons and jails, and this negatively affects clinicians’ and their delivery of care. Despite clinicians’ best efforts to improve healthcare in prisons and jails, participants described how the very structure and punitive model of these facilities limited their efforts. Even for those who found some success from their advocacy, this additional work led to moral distress and burnout, with some ultimately leaving the system. Most clinicians we spoke to chose to work in this environment to deliver care to a vulnerable population, not because they agree with the punitive nature of the carceral facilities. However, despite these intentions clinicians wrestled with feeling complicit in the system. We argue that the punitive purpose of the carceral system impacts all who enter the system, though those who are incarcerated are, of course, impacted the most. Our findings from this study demonstrate the need for additional research examining how the inherent structural violence of carceral institutions impacts clinicians who seek to provide care to incarcerated patients. Future research should also focus on interventions to mitigate this violence and support clinicians in carrying out their healthcare mission within these institutions.

Importantly, future research should center the experiences of clinicians in prisons and jails with marginalized identities, including clinicians who identify as BIPOC and LGBTQIA+, and how they navigate advocacy based on their positionality. Analysis in this space will be strengthened by a continued exploration of the role of power and oppression in healthcare in carceral spaces. While we have elucidated some forms of structural and cultural barriers to care, future research should seek to understand if or how clinicians engage in advocacy aimed at nonreformist reforms (Ben-Moshe 2020) that improve health care for the incarcerated population without strengthening the carceral system. As we have argued, prisons and jails are first and foremost institutions of punishment (Davis, 2003) and as Gawande (2006) argues, in these settings, medicine can become an “instrument of punishment.” An analysis grounded in theories of abolition may be useful to identify how clinicians view themselves within larger societal systems of oppression and how they balance their own positionality, the needs of the patients in front of them, and the larger system in which they work.

## Data Availability

The qualitative data collected for the study is not available for use by other researchers. Research participants did not consent to their data being shared beyond the study team.
